# Particle Filtering Based Remaining Useful Life Prediction for Electromagnetic Coil Insulation

**DOI:** 10.3390/s21020473

**Published:** 2021-01-11

**Authors:** Haifeng Guo, Aidong Xu, Kai Wang, Yue Sun, Xiaojia Han, Seung Ho Hong, Mengmeng Yu

**Affiliations:** 1 Key Laboratory of Networked Control Systems, Chinese Academy of Sciences, Shenyang 110016, China; guohaifeng@sia.cn (H.G.); xad@sia.cn (A.X.); sunyue@sia.cn (Y.S.); hanxiaojia@sia.cn (X.H.); 2 Shenyang Institute of Automation, Chinese Academy of Sciences, Shenyang 110016, China; 3 Institutes for Robotics and Intelligent Manufacturing, Chinese Academy of Sciences, Shenyang 110169, China; 4 University of Chinese Academy of Sciences, Beijing 100049, China; 5 Liaoning Institute of Science and Technology, Benxi 117004, China; 6 Department of Electronic Engineering, Hanyang University, Ansan 15588, Korea; shhong@hanyang.ac.kr (S.H.H.); ymm929@gmail.com (M.Y.)

**Keywords:** insulation degradation, insulation failure, inter-turn short, resonant frequency, PF, prognostics

## Abstract

Electromagnetic coils are one of the key components of many systems. Their insulation failure can have severe effects on the systems in which coils are used. This paper focuses on insulation degradation monitoring and remaining useful life (RUL) prediction of electromagnetic coils. First, insulation degradation characteristics are extracted from coil high-frequency electrical parameters. Second, health indicator is defined based on insulation degradation characteristics to indicate the health degree of coil insulation. Finally, an insulation degradation model is constructed, and coil insulation RUL prediction is performed by particle filtering. Thermal accelerated degradation experiments are performed to validate the RUL prediction performance. The proposed method presents opportunities for predictive maintenance of systems that incorporate coils.

## 1. Introduction

Electromagnetic coil is the energy conversion component of electromagnetic valve, motor, transformer, and other relevant equipment, and its insulation failure is prominent. The study from Oak Ridge National Laboratory [1] shows that over 50% of solenoid valve failures of nuclear power plants in U.S. were attributed to electromagnetic coil failures (e.g., coil open, coil short); in the field of automobiles, about 40% of motor failures are caused by stator insulation [2]; in the field of generators, 56% of generator failures are related to insulation failures [3]; according to the statistics in literature [4], the failures of transformer winding account for 70–80% of the total transformer failures, and among which the winding insulation failure rate is the highest. Therefore, it is important to study the online monitoring approaches for the insulation state of electromagnetic coils, this will benefit for the reliable operation and predictive maintenance of equipment and reducing the maintenance cost of factories.

Prognostics and health management (PHM) technology is one of the key technologies in smart manufacturing. PHM turned equipment management from traditional failure management into degradation management. Continuous and reliable operation of equipment are realized through predictive maintenance [5,6]. The accurate acquisition of health information of key components is the base for predicting the health status of the equipment. In this paper, the PHM technology based on key components is used to study the trend prediction for degradation status of electromagnetic coils.

The failure of electromagnetic coils is usually caused by the inter-turn insulation failure. The classic methods used to characterize the quality of coil insulation are based on visual inspection, electrical, physical, and chemical measurement. In the literature [7,8,9,10,11,12], offline detection methods based on temperature, current, neutral point voltage, magnetic flux leakage, and other parameters are introduced, and these methods can only detect the failures of electromagnetic coils, but they cannot detect the degradation process of insulation performance of electromagnetic coils. Considering that insulation failure usually occurs suddenly and has catastrophic effects, insulation degradation monitoring of the electromagnetic coil is preferred to enable predictive maintenance prior to a failure detection that could cause catastrophic damage. With the degradation of stator winding, twisted pair, and electromagnetic coil, F. Perisse and N.J.Jameson et al. [13,14,15,16,17,18] found that the parameters of parasitic capacitance, impedance, and reactance would change under high frequency conditions, and these characteristic parameters can be used to characterize the insulation degradation of electromagnetic coils, however, a large amount of data is needed to determine the parameters at a specific frequency which can assess the degradation of the solenoid. The process of insulation degradation is not systematically analyzed and modeled in the paper, and it is difficult to predict the degradation of the insulation performance of electromagnetic coils.

In this paper, the insulation performance degradation of electromagnetic coils is studied under the condition of constant high temperature. Through analyzing the insulation degradation mechanism and the data of insulation degradation of electromagnetic coils, resonance frequency is used as a characteristic parameter to characterize the degradation state of insulation. Based on the degradation state data, the insulation performance degradation model of electromagnetic coil is established. The insulation performance degradation prediction algorithm of electromagnetic coil is proposed based on particle filtering.

This paper is organized as follows. In Section 2, the failure mechanism of an electromagnetic coil in a high temperature environment is analyzed, and resonant frequency is selected as a health indicator for electromagnetic coil degradation monitoring based on the equivalent circuit model and lumped capacitance network analysis. The accelerated degradation experimental platform is described to obtain characteristic data. In Section 3, the model of insulation performance degradation is described with characteristic data collected through experiment. In Section 4, particle filtering based prediction algorithm is introduced for degradation of insulation performance of electromagnetic coil. In Section 5, the conclusion of this paper is given.

## 2. Framework for Coil Insulation Remaining Useful Life (RUL) Prediction Based on Data-Driven Methods

Prognostics and health management (PHM) is a method that permits the reliability of a system to be evaluated in its actual life-cycle conditions, and life prediction and health management are the core technologies in PHM. PHM methodology is based on monitoring parameters that are sensitive to impending failure precursors. The precursor is usually a change in a measurable parameter that can be associated with impending failure. Life prediction is considered as the foundation and core content of PHM, which can be roughly divided into a physical failure model based on the method and data-driven method. Since it is difficult to obtain the physical failure mechanisms, data-driven prediction methods have become mainstream techniques in recent years. The life prediction methods based on data-driven include the traditional methods based on failure data, the methods based on degradation data, and the methods based on multi-source data fusion. The data-driven method based on degradation data can obtain the characteristic parameters of equipment health state by analyzing the monitored data, and then realize the life prediction of equipment, which has been developed rapidly. It is helpful for accelerated fatigue test to obtain degradation data of the equipment quickly and effectively.

The electromagnetic coil, which is generally referred to as magnet wire, is constructed of conductor (usually copper) coated by insulation material (usually some kind of polymer). The electromagnetic coil is susceptible to coupled electrical/thermal failure mechanisms. When current is passing through the wire, Joule heating will cause the increase of wire temperature, this will lead the expansion of the conductor, placing mechanical and thermal stresses on the insulation material. The mechanical and thermal stresses then lead to insulation degradation and insulation failure.

**Figure 1:**
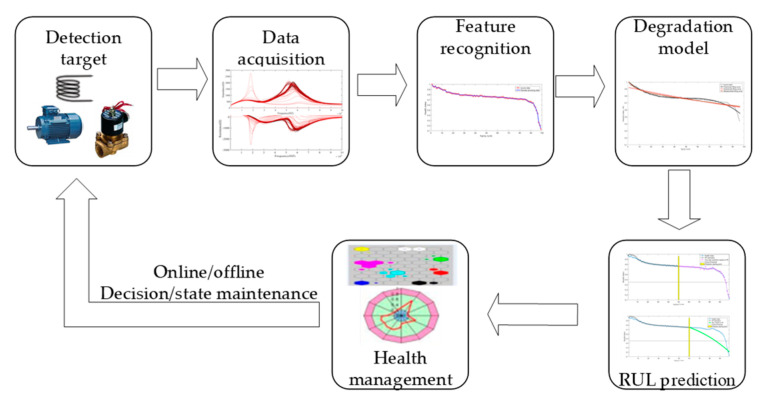
The prognostics and health management (PHM) implementation scheme of the electromagnetic coil.

Taking the electromagnetic coil as the research object, with the help of accelerated fatigue test, the whole life cycle data of the electromagnetic coil are collected; the parameters which can represent the degradation characteristic of the electromagnetic coil are determined by analyzing the collected data; the life degradation model of the electromagnetic coil is established based on the above characteristic parameters; then, the trend prediction and RUL prediction of the electromagnetic coil are realized, and the health status of the electromagnetic coil is analyzed; eventually, the health assessment and predictive maintenance are realized, as shown in Figure 1.

## 3. Electromagnetic Coils Data Acquisition Platform Construction and Data Analysis

### 3.1. Data Acquisition Platform Construction

**Figure 2:**
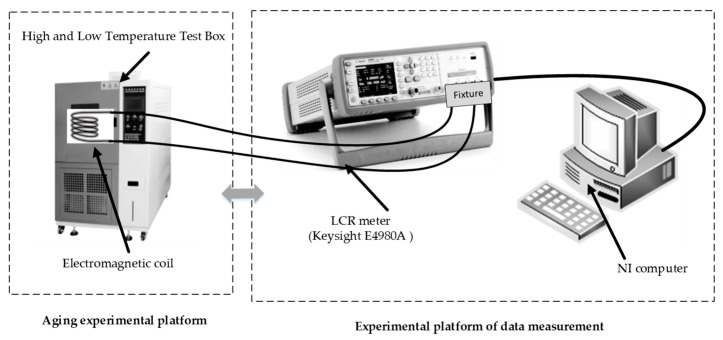
Experimental platform of the electromagnetic coil: Aging experimental platform is used for accelerated aging test at constant high temperature, experimental platform of data measurement is used to measure the electrical parameters of the electromagnetic coil.

In order to further study the degradation of electromagnetic coil insulation performance, the experimental platform is set up with constant high temperature stress, as shown in Figure 2. The experimental scheme is determined as follows: The electromagnetic coil was put into the chamber which is set at 220 °C, and the electrical data (inductance, resistance, impedance, reactance) could be collected by sweeping frequencies from 50 kHz to 1 MHz every 2 h, which is the frequency range provided by the LCR meter (Keysight’s E4980A inductance-capacitance-resistance); the electrical data are collected by NI computer; and direct-current resistance (DCR) of the coil was collected 8 times per cycle, and the average of DCR value is taken as the final value of DCR, which is used to determine whether the coil is in the degradation phase (there is no turn-to-turn short circuit in the coil) or failure stage during the aging test.

### 3.2. Data Acquisition and Analysis

**Figure 3:**
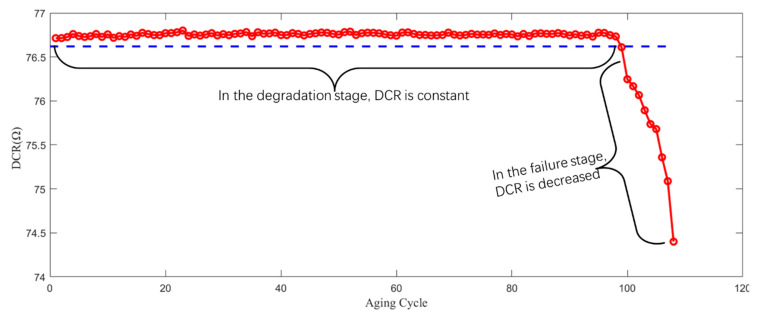
Measurement value of DCR of the electromagnetic coil during the aging experiment.

With the help of thermal acceleration experiment, the life cycle data of electromagnetic coil are obtained. In this experiment, the direct current resistance (DCR) of the electromagnetic coil used is 76.713 Ω, and its single turn DCR is 0.093 Ω. In the degradation process, the DCR of the electromagnetic coil is constant, there is no inter turn or inter layer short circuit in the electromagnetic coil, but in the failure process, the DCR decreases significantly, and there is an inter turn or inter layer short circuit, as shown in Table 1. During the acceleration experiment, the DCR of the electromagnetic coil is monitored in real time. As shown in Figure 3, the DCR of the electromagnetic coil remains unchanged before the 99th cycle, however, the data after 100 cycles show that the DCR decreases significantly (i.e., short circuit and hot spot formation), and then the failure leads to more hot spots and a more serious short circuit. When the DCR is detected to decrease, this indicates that the whole life data of the electromagnetic coil have been obtained.

**Figure 4:**
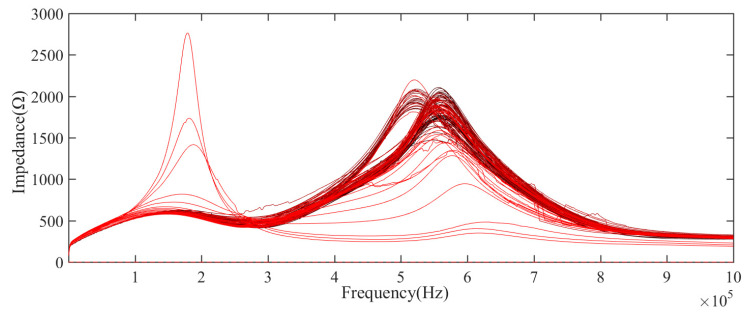
The measurement value of impedance spectra of the electromagnetic coil in the whole life.

**Figure 5:**
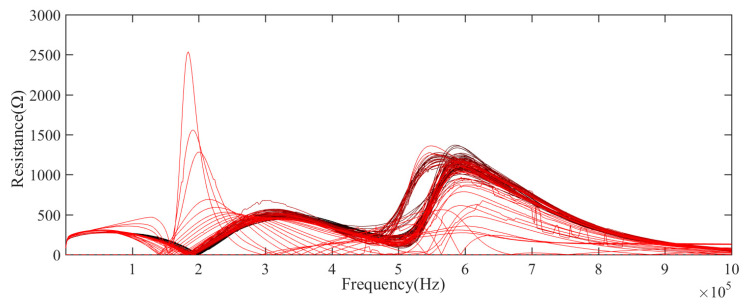
The measurement value of resistance spectra of the electromagnetic coil in the whole life.

**Figure 6:**
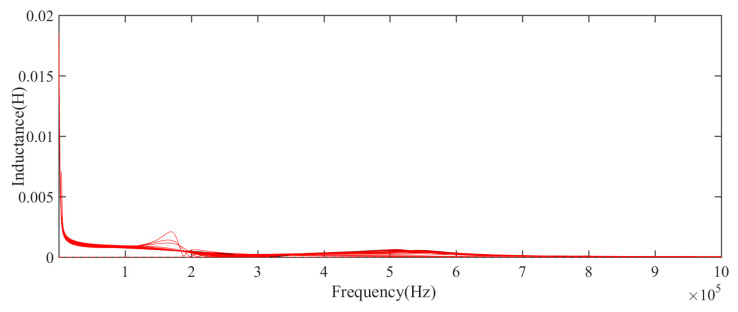
The measurement value of inductance spectra of the electromagnetic coil in the whole life.

**Figure 7:**
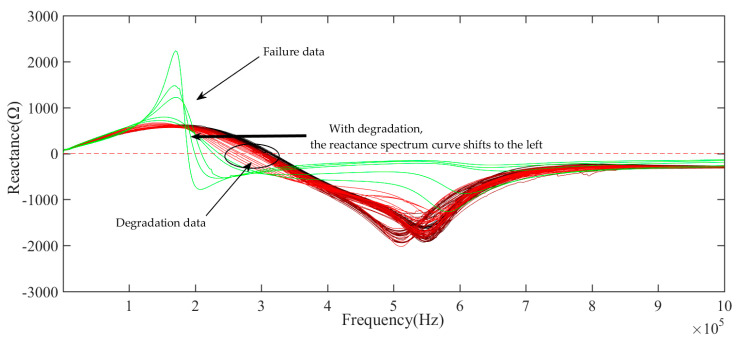
The measurement value of Reactance spectra of the electromagnetic coil with aging time.

LCR meter is used to collect impedance, resistance, inductance, and reactance data in a specific frequency range, as shown in Table 2. Figure 4, Figure 5 and Figure 6 show the impedance and resistance, inductance data of 106 aging cycles, in the degradation phase, the data of each cycle is not clearly distinguished, while in the failure phase, the three kinds of electrical data have significant changes compared with their respective data in the degradation phase. As shown in Figure 7, the reactance spectrum data of each aging period are collected. With the increase of aging time, when the electromagnetic coil is in heathy and degradation state, the reactance spectrum curves move from the right to the left regularly, the color of the curve gradually changes from black to red. After the 100th cycle, the reactance spectrum curve changes significantly (the green curve in Figure 7). Combined with DCR data, it indicates that the electromagnetic coil has been short-circuited.

## 4. Determination of Insulation Degradation Characteristic Parameters and HI Construction

The resonant frequency is the frequency at which reactance (imaginary part of impedance) is zero, that is, the inductive reactance is equal to the capacitive reactance. With the accelerated fatigue test, the resonance frequency has the decreasing gradually trend, as shown in Figure 7. According to the references [19,20], when the imaginary part is equal to zero, then the resonant frequency can be expressed as:

(1) $$f_{r}=\sqrt[{2}]{L/C_{g}-R^{2}}/L$$

where:

$f_{r}\;$is the resonant frequency.

According to the reference [19,20,21,22], with the degradation of electromagnetic coil, the R and inductance remain constant, and the $C_{g}$ changes with the thickness of insulation material, and then the resonance frequency also changes. Therefore, the resonance frequency, as an inherent index of electromagnetic coil, can be used to evaluate the health status of electromagnetic coil.

**Figure 8:**
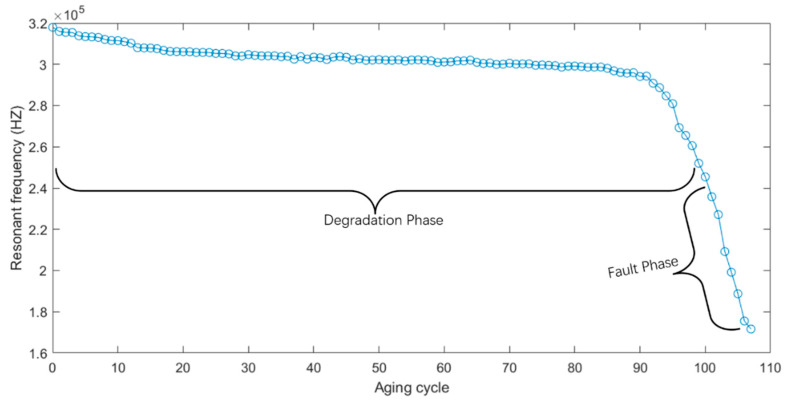
Degradation trend of resonant frequency of the electromagnetic coil.

According to formula (1), the resonance frequency will change with the degradation of the electromagnetic coil. The resonant frequency of each cycle can be used as an index to evaluate the health status of the electromagnetic coil. By fitting the reactance data collected from the experiment under multiple frequencies, the resonant frequency could be obtained under the current state. In the process of degradation, the resonance frequency generally shows a downward trend, cycle 1 to cycle 99 is the degradation phase, cycle 100 to cycle 106 is the failure phase, as shown in Figure 8. The test result demonstrates that the resonance frequency could be used as an effective characteristic parameter to evaluate the healthy status of the electromagnetic coil.

The health indicator (*HI*), which is homogenized based on resonant frequency, is introduced to indicate the degree of insulation degradation of the electromagnetic coil, as shown in formula (1). Therefore, *HI* is equal to 0.45, which is the failure threshold of the electromagnetic coil.

(2) $$HI\left(k\right)=\left(f\left(k\right)-f_{\mathrm{failure}}\right)/(f_{\mathrm{health}}-f_{\mathrm{failure}})$$

where:

$f_{\mathrm{health}}$ is resonant frequency of the health coil;

$f_{\mathrm{failure}}$ is resonant frequency of the failure coil; and

f(k) is the resonant frequency of cycle k.

## 5. Modeling of the Degradation of the Insulation Performance of the Electromagnetic Coils and RUL Prediction Based on Particle Filtering

### 5.1. Wavelet Threshold Denoising Method

**Figure 9:**
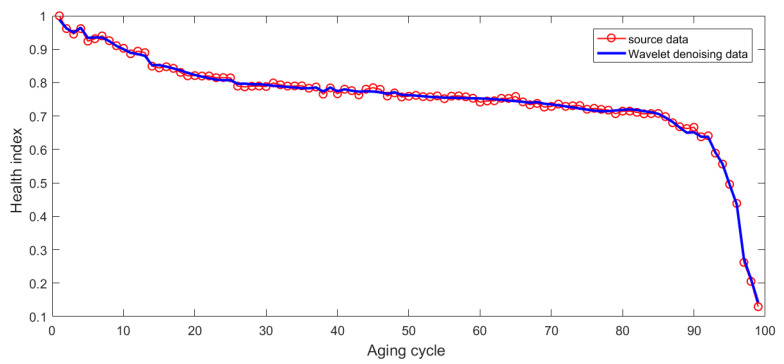
Degradation trend of health index of the electromagnetic coil after wavelet denoising.

In the accelerated degradation experiment, high-frequency data are collected. The accuracy of real data is undermined because of the interference of environmental conditions in the measurement process and the sensitivity of high frequency data. In order to obtain the high frequency data accurately, using filtering technology to filter out the noise interference is necessary. Wavelet denoising has a wide range of adaptability, and this will improve the accuracy of the data. After the use of the wavelet de-noising method, the degradation curve based on health index is obtained. It shows that the trend of the degradation curve is more obvious, which is more beneficial for the enhancement of the accuracy of degradation trend prediction and RUL prediction, as shown in Figure 9.

### 5.2. Life Model Based on Health Index

The main idea of degradation modeling is to describe the distribution of degradation trajectory with the data of characteristic parameters. In this paper, degradation prediction research of long-life equipment is carried out by studying the change of its characteristic parameters. Through the characteristic data, which can represent the trend of the equipment life, the degradation model based on the characteristic parameters is established to predict the health status of the equipment. Several common degradation models are shown in formula (3)–(5):

(3) HI(k)=a∗exp(b∗k)

(4) HI(k)=a∗exp(b∗k)+c∗exp(d∗k)

(5) $$HI\left(k\right)=a*k^{3}+b*k^{2}+c*k+d$$

where:

a、b、c、d is parameter of model;

k is aging time (measurement cycle); and

HI(k) is the health indicator of k measurement cycle.

**Figure 10:**
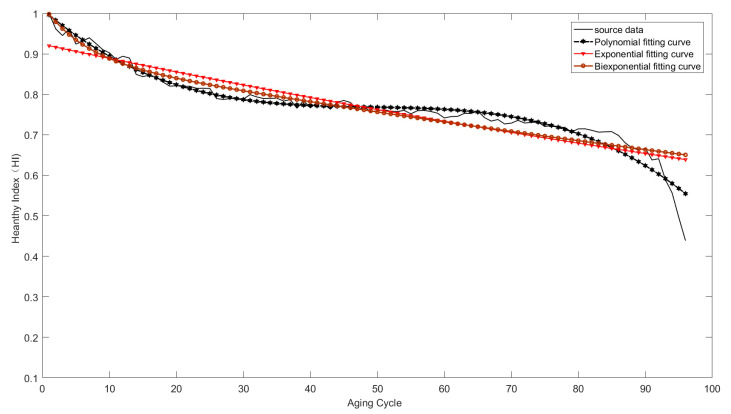
Curve-fitting model for the relationship between health indicator and aging time.

Polynomial and exponential model (formula (3)–(5)) are used to fit the life cycle data of the electromagnetic coil, and the results are shown in Figure 10. The results show that there was a decreasing trend of the health indicator as the coil aged during the degradation phase. The curve-fitting model, which can accurately describe the performance degradation trend, is identified as the degradation model of the electromagnetic coil. It is not expected to the two curve-fitting models (the formula (3) and (4)) that can’t track the degradation data very well, but the polynomial model (formula (5)) is better than the first two models, as shown in Figure 10. Therefore, the polynomial model is used as the life degradation model of the insulation performance of the electromagnetic coil, and the coefficients of the polynomial degradation model are obtained by means of the fitting tool of MATLAB.

### 5.3. Particle Filtering

Particle filter (PF) algorithm is a non-linear, non-Gaussian system filtering method based on the Monte Carlo idea, UKF, and other Kalman filters that adopt the statistical linearization method, which is based on the linear and Gaussian assumption of the system. When the system is in nonlinear and non-Gaussian state, the prediction effect of UKF is not obvious. The research object of this paper is an electromagnetic coil with a long life, and the industrial environment is complex, and its life distribution is non-linear and non-Gaussian, so the PF with wider applicability is selected. Therefore, the PF algorithm is one of the effective methods to realize the trend and RUL prediction based on model [23]. The two important steps of PF are: (1) Random particles are extracted from the empirical conditional distribution; (2) the weight of each particle is calculated according to the observation probability distribution, importance distribution, and Bayesian formula. As the number of samples increases, the method approaches to the real posterior probability density function of state variables, and then the prediction of the degradation trend and RUL of the electromagnetic coil is realized. The model based on particle filter can be established by formula (6):

(6) $$\{\begin{array}{c} x_{k}=f\left(x_{k-1}\right)+u_{k}\\ y_{k}=h\left(x_{k}\right)+v_{k}\; \end{array}$$

where:

$x_{k}\;$is the state vector;

$y_{k}\;$is the observation vector;

$f\left(x_{k-1}\right)$ is state transition function;

$h\left(x_{k}\right)$ is observation function;

k is the time index;

$u_{k}$ is process noise sequence, the gaussian distribution with mean value of 0 and variance of σ^2^ is satisfied; and

$v_{k}$ is observation noise sequence, the gaussian distribution with mean value of 0 and variance of σ^2^ is satisfied.

**Figure 11:**
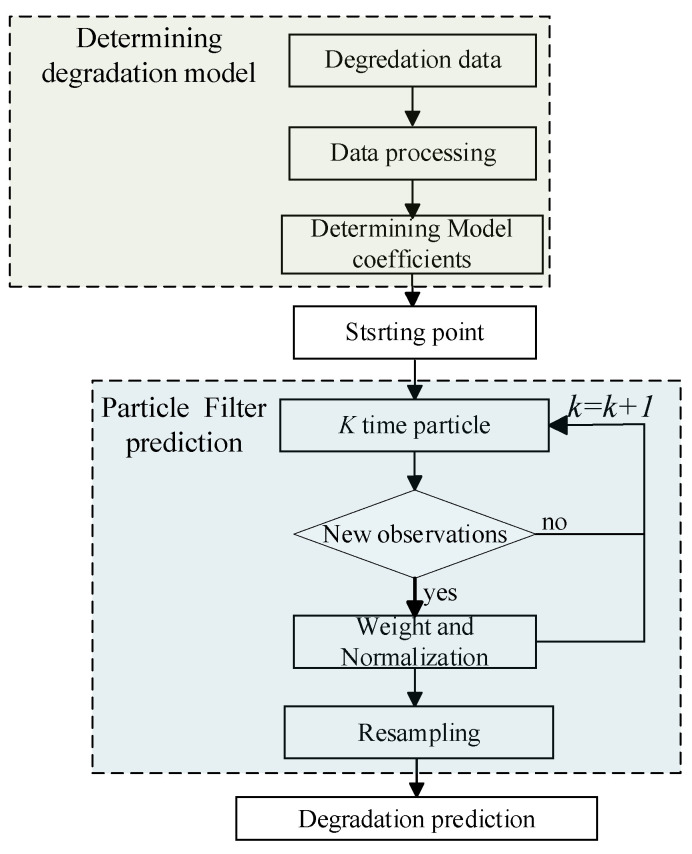
Prediction flow chart based on particle filtering.

Each particle of PF algorithm is a set of modified parameters, and the particles are carried out at the same time, therefore, the PF algorithm is a multi-point parallel algorithm, which greatly improves the prediction accuracy. The following Figure 11 is the prediction flow chart of particle filtering algorithm. In this paper, the degradation model is used as the observation equation, and the health indicator of the electromagnetic coil is used as the observation.

### 5.4. Prediction of Insulation Degradation Based on Particle Filtering Algorithm

In the process of degradation of insulation performance of the electromagnetic coil, the data collection process contains white noise with zero mean and unknown variance. The state transition equation describes the functional relationship between the state of the system at the previous moment and the state at the current moment. Therefore, the degradation model of the insulation performance of electromagnetic coils (Formula 5) is needed to deal with the following forms:

(7) $$HI\left(k\right)=HI\left(k-1\right)+\left(3*a+b\right)*k^{2}+\left(3*a+2*b+c\right)*k+\left(a+b+c\right)$$

where:

HI(k) is k time health indicator;

HI(k−1) is k−1 time health indicator;

k is degenerate cycle (a cycle is 2 h); and

*a*, *b*, and *c* are the parameters of the degradation model.

With (7), the following formula can be obtained by integrating the upper constant term:

(8) $$HI\left(k\right)=HI\left(k-1\right)+\alpha *k^{2}+\beta *k+\gamma$$

where:

α=3∗a+b;

β= 3∗a+2∗b+c; and

γ=a+b+c.

It can be seen from the above formula that the health indicator at k-time is composed of the above two items, which are related to both the health index at k–1 time and the degeneration cycle. Therefore, the degradation model of the electromagnetic coil is taken as the observation, and the parameters of this model are taken as the state variables, as shown in formula (9):

(9) $$\{\begin{array}{c} \alpha \left(k\right)=\alpha \left(k-1\right)+u_{\alpha }\;\\ \beta \left(k\right)=\beta \left(k-1\right)+u_{\beta }\;\\ \gamma \left(k\right)=\gamma \left(k-1\right)+u_{\gamma }\;\\ HI\left(k\right)=HI\left(k-1\right)+\alpha *k^{2}+\beta *k+\gamma +v_{k} \end{array}$$

**Figure 12:**
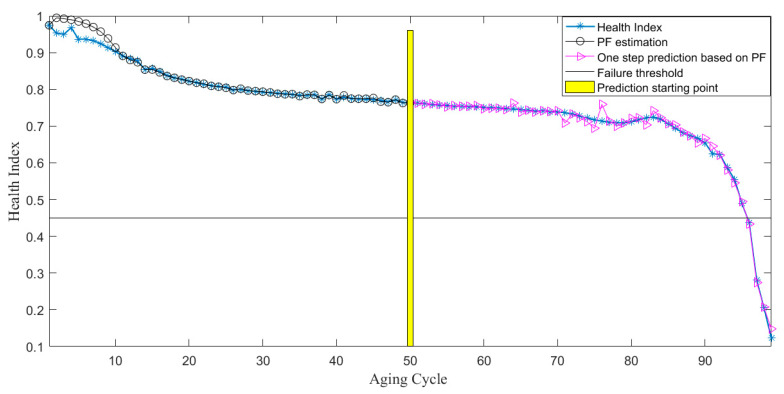
Prediction of particle filtering algorithm at the beginning of cycle 50 of the electromagnetic coil.

The prediction starting point is set to be Start = cycle 50. As shown in Figure 12, the prediction basically reflects the degradation trend of the insulation of the electromagnetic coil, which has a certain guiding role for the insulation degradation of the electromagnetic coil.

### 5.5. RUL Prediction of the Electromagnetic Coil Based on Particle Filter

The above experiments and analysis show that the *HI* can describe the performance degradation of the electromagnetic coil, and the 45% decrease of the *HI* is taken as the failure threshold of the electromagnetic coil. The RUL prediction of electromagnetic coil is realized by the particle filter mentioned above, that is, the life is terminated when the *HI* is equal to failure threshold, and the number of cycles from the predicted starting point to the failure threshold is regarded as the RUL. In order to evaluate the accuracy of RUL, the absolute error formula of RUL is defined as follows:

(10) $$RUL_{error}=\left|RUL_{true}-RUL_{prediction}\right|$$

**Figure 13:**
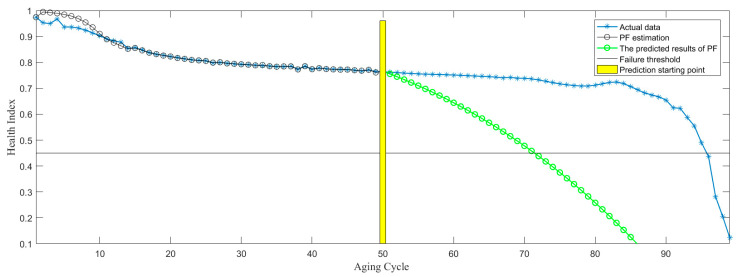
RUL (remaining useful life) prediction based on starting point 50 cycle.

**Figure 14:**
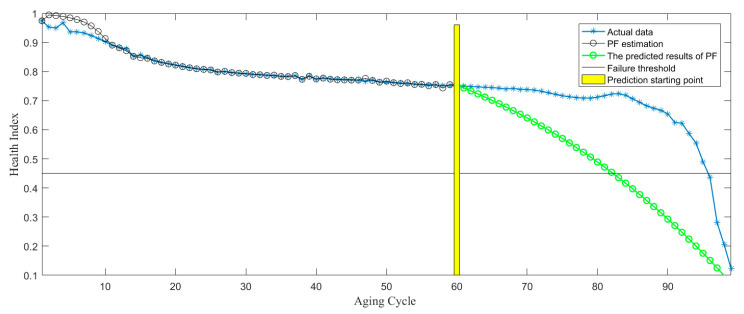
RUL prediction based on starting point 60 cycle.

**Figure 15:**
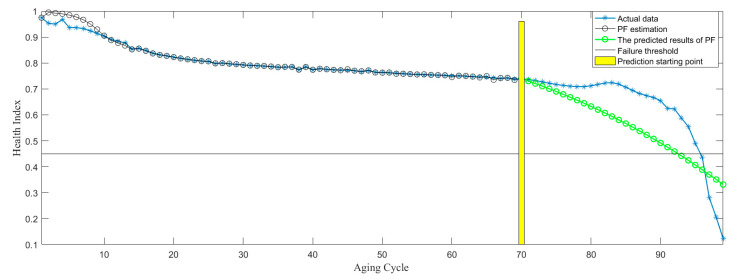
RUL prediction based on starting point 70 cycle.

In the process of RUL prediction, the prediction starting point is set to be Start = cycle 50, 60, and 70, as shown in Figure 13, Figure 14 and Figure 15. According to the above three RUL prediction results, the prediction error of RUL is quantitatively given by equation 11, as shown in Table 3. It can be shown from the above results that RUL prediction of the electromagnetic coil can be realized by PF algorithm, meanwhile, with the increase of the historical data, the accuracy of RUL prediction is further improved. However, if there is no large amount of historical data, it is difficult to predict the remaining life, and it lacks accuracy, as shown in Figure 13. Only when a lot of historical data is provided, the RUL prediction based on data-driven can be more accurate. However, the RUL prediction based on the physics-of-failure model can be realized without a lot of data. At present, our research group is studying the physics-of-failure model of the electromagnetic coil based on creep degradation, and the subsequent establishment of the physics-of-failure model can solve the problem due to lack of data.

## 6. Conclusions

The electromagnetic coils are the widely used components in many applications and systems. Their insulation is failure-prone, which can lead to disastrous consequences. Therefore, predicting RUL for electromagnetic coil insulation can effectively avoid the unexpected shut-down and thus improve the availability of the related equipment that incorporate coils.

Data-driven PHM framework is proposed to perform insulation degradation monitoring and RUL prediction for coil insulation. Coil electrical parameters, which include DCR and impedance under different frequencies, were collected by thermal accelerated fatigue test. Coil resonant frequency is identified as the insulation degradation characteristic parameter by analysis of the insulation failure mechanism and collected experimental data. The insulation health indicator is thus defined based on resonant frequency to indicate the health status of the electromagnetic coil. Further, the life model of the electromagnetic coil is constructed based on the polynomial fitting for resonant frequency data. The RUL prediction of electromagnetic coils are realized by PF. In order to increase the accuracy and reduce the complexity of particle filter, the polynomial model with one parameter reduced is used as the observation equation of the PF algorithm. The prediction accuracy of RUL is gradually improved with the increase of historical data. The prediction of electromagnetic coil health status provides support for predictive maintenance of the related equipment that incorporates coils.

In the future research, the accelerated fatigue test of the electromagnetic coil will be carried out to collect the degradation data under different fatigue temperature conditions. The degradation model of the electromagnetic coil is constructed under multi-temperature, which lays the foundation for the prediction of RUL on variable temperature environments on site. Meanwhile, the physics-of-failure model based on creep degradation is established, which solves the defect that RUL prediction relies seriously on historical data. Combined with the self-learning function of neural networks and the ability to find the optimal solution at high speed, the degradation model based on neural network is established to realize the RUL prediction.

## Figures and Tables

**Table 1:** Part of direct-current resistance (DCR) collected by LCR meter.

Cycle	DCR1	DCR2	DCR3	DCR4	DCR5	DCR6	DCR7	DCR8	DCR (Average)
1	76.71	76.71	76.71	76.71	76.71	76.71	76.71	76.71	76.71
10	76.71	76.71	76.71	76.71	76.71	76.71	76.71	76.71	76.71
20	76.71	76.71	76.71	76.71	76.71	76.71	76.71	76.71	76.71
30	76.71	76.71	76.71	76.72	76.71	76.70	76.71	76.71	76.71
40	76.72	76.71	76.72	76.72	76.71	76.72	76.72	76.72	76.72
50	76.72	76.72	76.722	76.72	76.72	76.72	76.72	76.72	76.72
60	76.72	76.71	76.72	76.71	76.72	76.72	76.71	76.72	76.72
70	76.72	76.71	76.72	76.72	76.72	76.72	76.71	76.72	76.72
80	76.71	76.71	76.71	76.71	76.71	76.70	76.72	76.72	76.71
90	76.71	76.71	76.71	76.72	76.71	76.72	76.72	76.71	76.71
100	76.62	76.62	76.62	76.63	76.63	76.63	76.64	76.63	76.63
106	74.35	74.35	74.37	74.34	74.35	74.35	74.36	74.33	74.35

**Table 2:** Part of the data collected by LCR meter under the frequency of 314,050 Hz.

Cycle	Acquisition Time	Frequency (Hz)	Impedance (Ω)	Resistance (Ω)	Inductance (H)	Reactance (Ω)
1	2020-4-28 08:02:45	3.14 × 10^5^	4.98 × 10^2^	4.97 × 10^2^	3.4 × 10^−5^	2.85 × 10^1^
10	2020-4-29 01:37:15	3.14 × 10^5^	4.65 × 10^2^	4.63 × 10^2^	3.5 × 10^−5^	−4.92 × 10^1^
20	2020-4-29 19:53:08	3.14 × 10^5^	5.39 × 10^2^	5.28 × 10^2^	5.7 × 10^−5^	−1.11 × 10^2^
30	2020-4-30 20:01:41	3.14 × 10^5^	5.51 × 10^2^	5.38 × 10^2^	6.1 × 10^−5^	−1.67 × 10^2^
40	2020-5-1 16:13:13	3.14 × 10^5^	4.45 × 10^2^	4.41 × 10^2^	3.2 × 10^−5^	−6.36 × 10^1^
50	2020-5-4 15:41:46	3.14 × 10^5^	4.56 × 10^2^	4.52 × 10^2^	3.0 × 10^−5^	−5.91 × 10^1^
60	2020-5-5 21:28:30	3.14 × 10^5^	5.68 × 10^2^	5.53 × 10^2^	6.5 × 10^−5^	−1.28 × 10^2^
70	2020-5-7 17:37:42	3.14 × 10^5^	4.57 × 10^2^	4.52 × 10^2^	3.6 × 10^−5^	−7.03 × 10^1^
80	2020-5-9 20:35:33	3.14 × 10^5^	4.71 × 10^2^	4.56 × 10^2^	5.9 × 10^−5^	−1.15 × 10^2^
90	2020-5-11 00:46:51	3.14 × 10^5^	5.45 × 10^2^	5.13 × 10^2^	6.4 × 10^−5^	−1.84 × 10^2^
100	2020-5-12 00:28:57	3.14 × 10^5^	5.26 × 10^2^	2.62 × 10^2^	2.3 × 10^−5^	−4.56 × 10^2^
106	2020-5-12 13:33:31	3.14 × 10^5^	3.07 × 10^2^	6.06 × 10^1^	1.5 × 10^−5^	−3.01 × 10^2^

**Table 3:** Prediction error analysis.

Staring Point	Real Life (Cycle)	Prediction Result (Cycle)	Absolute Error (Cycle)
50	96	72	24
60	96	83	13
70	96	93	3

## Data Availability

The data presented in this study are available on request from the corresponding author. The data are not publicly available due to the need for a short confidentiality period.
